# Lateral specialization in unilateral spatial neglect: a cognitive robotics model

**DOI:** 10.1007/s10339-016-0761-x

**Published:** 2016-03-28

**Authors:** Daniela Conti, Santo Di Nuovo, Angelo Cangelosi, Alessandro Di Nuovo

**Affiliations:** Department of Education Sciences, University of Catania, Via Biblioteca 4, 95124 Catania, Italy; Psychology Operative Unit, IRCCS “Maria SS” Oasi di Troina, 73, Conte Ruggero, 94018 Troina, Italy; Centre for Robotics and Neural Systems, Plymouth University, Drake Circus, Plymouth, PL48AA UK; Sheffield Robotics, Sheffield Hallam University, Howard Street, Sheffield, S11WB UK; Department of Engineering and Architecture, University of Enna “Kore”, Viale delle Olimpiadi, 94100 Enna, Italy

**Keywords:** Unilateral spatial neglect, Embodied cognition, Cognitive robotics, Hemisphere specialization, Neuropsychology

## Abstract

In this paper, we present the experimental results of an embodied cognitive robotic approach for modelling the human cognitive deficit known as unilateral spatial neglect (USN). To this end, we introduce an artificial neural network architecture designed and trained to control the spatial attentional focus of the iCub robotic platform. Like the human brain, the architecture is divided into two hemispheres and it incorporates bio-inspired plasticity mechanisms, which allow the development of the phenomenon of the specialization of the right hemisphere for spatial attention. In this study, we validate the model by replicating a previous experiment with human patients affected by the USN and numerical results show that the robot mimics the behaviours previously exhibited by humans. We also simulated recovery after the damage to compare the performance of each of the two hemispheres as additional validation of the model. Finally, we highlight some possible advantages of modelling cognitive dysfunctions of the human brain by means of robotic platforms, which can supplement traditional approaches for studying spatial impairments in humans.

## Introduction

*Unilateral Spatial Neglect* (USN) comprises a collection of behavioural symptoms in which patients appear to be incapable to perceive stimuli in spatial locations contralateral to the damaged cerebral hemisphere, e.g. after stroke (Heilman et al. 1994; Karnath et al. 2002). USN is also referred to as “visual neglect”, “hemispatial neglect” or “hemineglect”, and it is typically associated with damage to the Posterior Parietal Cortex (PPC), although, in many patients, lesions can be more extensive and involve also the premotor cortex (PMC). USN is a pathological condition that is more frequent, longer lasting, and more severe following lesions to the right hemisphere, RH, than to the left hemisphere, LH (De Renzi 1982; Plummer et al. 2003). As result, patients with neglect commonly ignore objects on the left side of space, fail to eat from the left side of the plate, and may dress the right side of the body only.

Most contemporary views of the neglect syndrome consider it to be a heterogeneous condition consistent with the heterogeneous nature of the associated lesion sites. The neglect emerges as a result of a combination of component cognitive deficits that may vary across patients and need not be neglect specific (Parton et al. 2004). Pouget and Driver (2000) theorized that USN is a selective loss of neurons representing particular locations in space for particular functions.

Indeed, USN is far from a unitary phenomenon and has been shown to fractionate into a number of dissociable components in terms of sensory modality, spatial domain, response laterality, motor output, and stimulus content (Barbieri and De Renzi 1989; Robertson and Halligan 1999). Furthermore, different USN disorders may exist, which may require type-specific rehabilitation approaches. This may have implications for epidemiological studies and for the development of new treatments. Theoretically driven epidemiological studies are required before adequately powered randomized controlled trials of rehabilitation can be conducted (Bowen et al. 1999). Given the complexity of the disease, various tools are needed to be able to diagnose the presence and the relative degree of impairment of the areas involved. This is crucial also for the patient’s rehabilitation. The most direct approaches to explore spatial impairments are neurophysiological studies in animals (e.g. Gottlieb et al. 1998), neuroimaging and lesion studies in humans (e.g. Parasuraman and Yantis 1998). Computer simulations can supplement these methods by testing hypotheses about the normal and disordered function of attentional processes. Computational models enable experimenters to make explicit assumptions and hypotheses, and to implement only the portions of the brain that need more focus. Moreover, the analysis of results can be conducted at a level of detail which would be difficult to achieve in other domains of cognitive neuroscience. This “ecological”, or Artificial Life approach adds further power to the connectionist modelling by means of simulating not only the brain and the nervous system, but also the body and the environment of artificial organisms (Langton 1995; Parisi et al. 1990).

Previous research (e.g. Cohen et al. 1994; Mozer et al. 1997) has shown that computational models of neglect can reveal emergent behaviours that are beyond the typical scope of speculating with non-computational models. Mozer and Behrmann (1990) “lesioned” an existing computational model of visual perception and selective attention called MORSEL (Mozer 1991) in accordance with the damage that was hypothesized to occur in the brains of neglect patients. The damaged model was then used to simulate some puzzling aspects of the performance of patients with neglect dyslexia (a reading disorder associated with neglect). Similarly, Lanyon and Denham (2010) examined the effects of a parietal lesion in their model of visual attention and search that is based on neurobiological evidence from monkey electrophysiology (Lanyon and Denham 2004).

Theoretical models of visual neglect can be usually divided into approaches based on an attentional or a representational account of the syndrome. An attentional account (e.g., Chatterjee 2003) considers neglect as a deficit in orienting visual attention to the affected hemispace, whereas a representational account interprets neglect as the result of impairment of one side of a particular spatial representation. Deco and Zihl (2004) presented an attentional model that was based on the ‘‘biased competition hypothesis’’ (Desimone and Duncan 1995). Spatial and object attention are accomplished by a multiplicative gain control that emerges dynamically through an inter-cortical mutual biased coupling. By damaging the model in different ways, authors report a variety of dysfunctions associated with visual neglect that can be simulated and explained as disruption of specific subsystems. In particular, authors were able to explain the asymmetrical effect of spatial cueing on neglect, and the phenomenon of extinction in the framework of visual search. Pouget and Sejnowski (2001) presented a representational model that can account for several behaviours shown by patients with hemi-neglect. In this model, contralateral neglect arises because the unilateral parietal lesions lead to a neuronal gradient in basis function maps producing an imbalance in the salience of stimuli that is modulated by the orientation of the body in space. Monaghan and Shillcock (2004) reported the results of a series of artificial neural network simulations of the line-bisection task that emphasized the hemispheric asymmetries in neglect cause and in its effects. They claimed that a model with neuro-anatomically realistic principles of connectivity in the nervous system could produce emergent behaviours that capture a wide range of quantitative and qualitative data observed in neglect patients.

Recent research suggests that spatial cognition models should be embodied (Coello and Delevoye-Turrell 2007; Trafton and Harrison 2011) and, in particular, some empirical data in cognitive neurosciences with USN patients (Richard et al. 2004; Saj et al. 2006) support this view showing that the general spatial processing is influenced by a distorted representation of the body, which is shifted in the direction of the lesion. Meanwhile, many projects in robotics and artificial intelligence have highlighted the value of a direct sensory-action approach where intelligence requires a body (Chaminade and Cheng 2009; De La Cruz et al. 2014; Di Nuovo et al. 2013; Fischer and Coello 2016; Levesque and Lakemeyer 2008), as opposed to classical intelligence which used the sensory-thought-action framework and involved a strong dissociation between the body and mind. But, so far, at the best of our knowledge, no other cognitive robotics model has been designed and applied to study USN.

In this paper, we present a novel artificial neural network model to control the spatial attention of the iCub robotic platform from proprioceptive information including not only visual information but also motor inputs. The architecture is designed to model the RH specialization for elaboration of the visuo-spatial information, which emerges naturally because the network initialization incorporate some mechanisms inspired by the plasticity of the human brain (Gould et al. 1999).

The model is studied and validated by replicating an experiment that was carried out with human patients affected by USN (Bisiach et al. 1985), which addresses the question of bodily reference system of space representation. The model links are damaged to simulate different USN conditions and tested in a manipulation task that requires the cognitive robot to perform a spatial exploration. The experiments aim to confirm the validity of the hemisphere specialization and to examine the relation of unilateral neglect to the sagittal mid-plane of the trunk and the line of sight. Finally, rehabilitation sessions are simulated to see the recovery capability of the network.

Details of the model and of the experimental setup are in Sect. “Materials and methods”. Section “Experimental results and discussion” reports and discusses the numerical results of the experiment with the iCub robot. Finally, Sect. “Conclusion” gives our conclusion.

## Materials and methods

### The iCub robotic platform and the neural network architecture

**Fig. 1:**
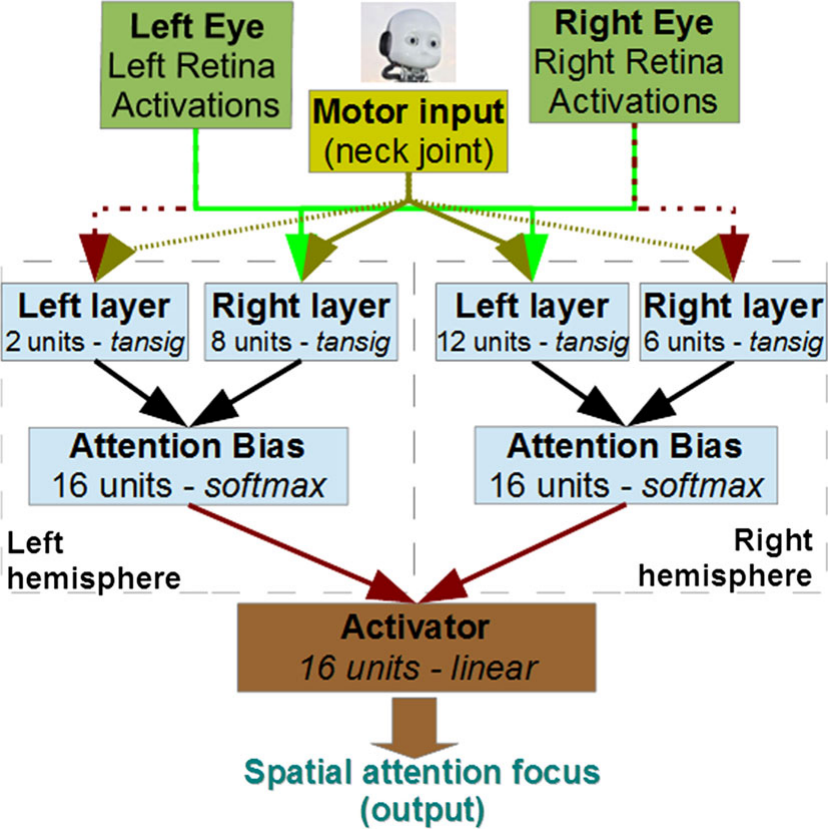
The neural network model for simulation of USN. The hidden layers are divided into two regions to mimic the separation of the cerebral hemispheres. The number of units and transfer functions used to implement the neural processing are specified for each layer. Connections from Attention Bias to Cognition (*red lines*) are cut to simulate the hemisphere damage. In the control experiment, dotted lines are removed and layers have the same number of units. In the second experiment, the RH has stronger connection weights and more neuronal units (as reported in Figure 1) to simulate plasticity and prompt the emergence of the hemisphere specialization for processing visuospatial information (color figure online)

The robotic model used for the experiments is the iCub humanoid robot, which is a child-like humanoid robot platform designed to facilitate developmental robotics research (e.g. Metta et al. 2010). The robot is controlled by an artificial neural network architecture, which is schematically represented in Fig. 1. The model has few functions of the PPC, which is thought to play a crucial role in the computation of sensorimotor transformations and in linking sensation to action.

The hidden layers are divided into two regions to mimic the separation of the cerebral hemispheres. The object positions were calculated from pictures taken by the eye cameras during the training phase. These positions were represented as a 2D pixel matrix, and they are the input of our artificial neural architecture (*target inputs*). The other (motor) input is the neck joint angle. Input coordinates were different for the RH and LH as they were retrieved using, respectively, the right and left eye cameras pictures, this way the coordinates were relative to the camera position. To simulate the antagonist action of the real human neck muscle, we coded the right input as the opposite of the left values: if the neck was turned 40 degrees to the right, the right motor input was −40 (means that the right muscle was flexed), at the same time, the left motor input was 40 (means that the left muscle was extended); meanwhile, if the neck was turned 40° to the left, the right motor input was 40 (means that the right muscle was extended), at the same time, the left motor input was −40 (means that the left muscle was flexed).

The attention bias layers use the *softmax* function to calculate the unit activation:

$$ {\text{softmax}}\left( {\varvec{q},i} \right) = \frac{{e^{{q_{i} }} }}{{\mathop \sum \nolimits_{j = 1}^{n} e^{{q_{j} }} }} $$

where the vector **q** is the net input to a *softmax* node, and *n* is the number of nodes in the *softmax* layer. The *softmax* function produces outputs that are real values in the range [0, 1] that sum up to 1, which can be also interpreted as probabilities.

The role of the final layer (Activator) is to simulate the final processing of the attentional biases and to produce the final output that will activate the action associated with one target area. The activator has a linear transfer function that combines the activation from LH and RH and generates the final classification likelihood of the sixteen possible target positions. In this paper, we refer to the final output as the likelihood, which can be defined as how likely it is to perform the action to explore a specific target area on the table. Note that the final output activation can be greater than 1 or lower than 0 as it is the combined result of the sum of the LH and RH activations.

Finally, to model the asymmetries between the two hemispheres, we incorporate in our model the following plasticity mechanisms that a stronger activity on the RH should prompt (e.g. Pascual-Leone et al. 2005):

1. the reinforcement of the intra-hemispheric connections;
2. the formation of new pathways.

In practice, when we model the RH specialization in our architecture (see Fig. 1 for details):

1. the stronger links are modelled via the initialization of the LH connection weights in a smaller range, i.e. between −0.1 and 0.1, while the RH connection weights are greater (e.g. in the standard range [−1, 1]);
2. the new pathways are modelled allocating four additional neural units to the RH layers. This way, in our experiments the relevant specialization emerges naturally after the backpropagation training.

### The experimental setup and procedure

**Fig. 2:**
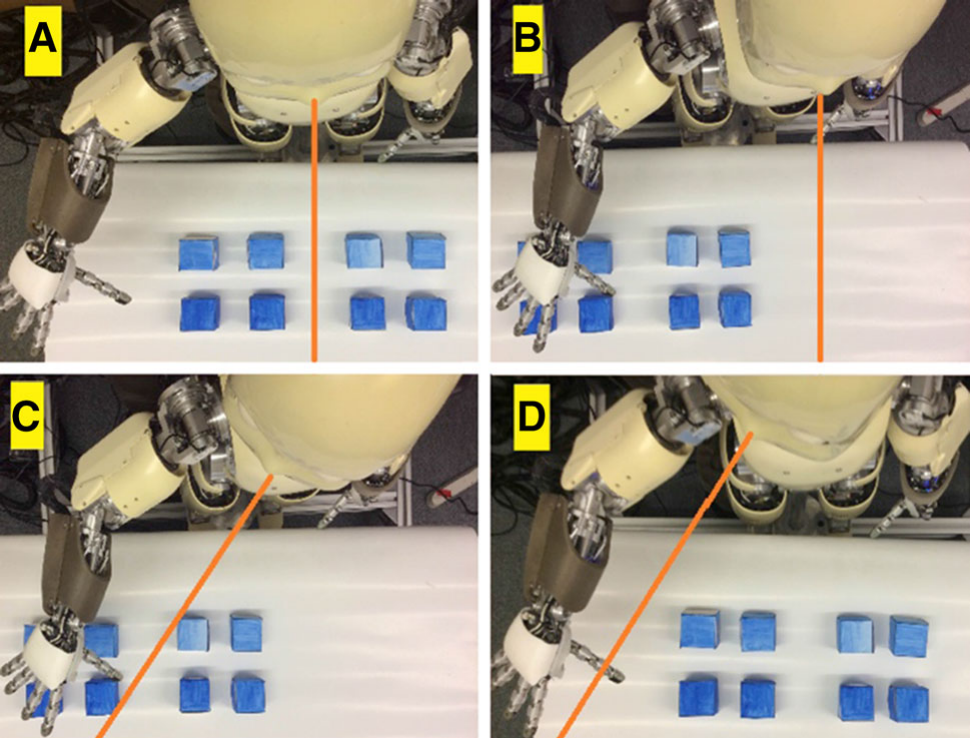
The four experimental conditions. The *orange lines* highlight the head axes. In conditions **a**, **b**, they are right in front of the robot while in conditions **c**, **d** the head is turned 40° to the right (color figure online)

The model presented in this paper is validated through experimental tests that resemble a previous study with human patients. USN patients repeated a manipulation task in four different conditions for placing targets and for orienting longitudinal axes of the head and eyes (Bisiach et al. 1985). To this end, we set up the four conditions as represented in Fig. 2 using the iCub robot. In condition (A), the eight targets were placed in front of the robot, so that the longitudinal axes of the head and of the exploring hand lay in the sagittal mid-plane of the trunk, while the eyes looked straight ahead. In condition (B) the targets were (displaced in such a way that they all were) on the right of the sagittal mid-plane of the trunk, while the head and eyes were kept at 0°. In condition (C) the targets remain as in (B), but the neck joint was rotated so that head and eyes were at an angle of 40° with respect to the sagittal mid-plane of the trunk. Finally, in condition (D), targets were returned to their original position as in (A), while head and eyes were kept at an angle of 40°, as in (C).

**Fig. 3:**
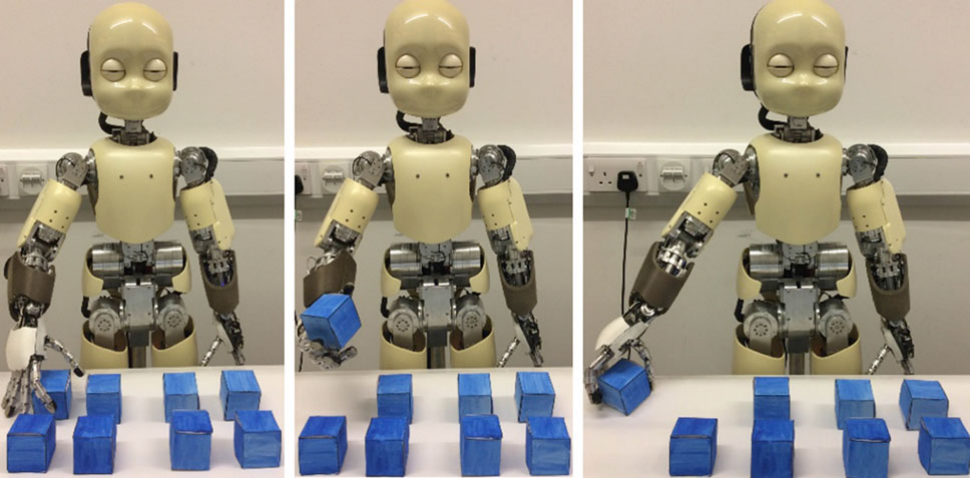
The experimental task: the iCub robot removes an object from the working area

For each condition, targets were eight small blue cubes placed on the table in two rows of four. The experimental task for the robot was to explore one by one the eight positions and to remove the objects placed on the table, without visual control (see Fig. 3 for an example).

In a preliminary phase, the robot was trained to accomplish the experimental task using a pre-programmed routine. To train the network we applied the gradient descent with momentum backpropagation algorithm (Rumelhart et al. 1986), which is the most widely used method for training feedforward neural networks. For each iteration of the backpropagation algorithm, called epoch, the batch training procedure is applied and all the examples in the training set are inputted to the network before the weights are updated.

The goal of the training was to associate the action routine with the spatial attentional focus that identifies a specific place in the table. The action primitives needed to perform the task were previously learned by the robot. The model was trained using all the possible target positions on the table. A total of sixteen positions were identified, equally distributed on the left and on the right side of the robot in order to have a balanced training scenario that covers the entire attentional field.

In the lesioning experiments, we simulated damages in different parts of the artificial hemisphere by cutting neural links (i.e. assigning 0 to connection weights), obtaining also an intra-hemispheric disconnection between anterior and posterior layers. A similar approach was also found to yield neglect-related behaviour in previous simulation studies (e.g. Di Ferdinando et al. 2007; Mozer 2002).

Finally, as a further experiment, we re-applied the backpropagation algorithm to simulate a rehabilitation therapy and the recovery after the damage as additional validation of the model. Every session comprised 100 applications (epochs) of the backpropagation algorithm, and we repeated the experiment and recorded the omissions. In this scenario, the results are analysed in terms of the number of sessions needed to recover and the performance of the two hemispheres is compared.

In this case, the supervised backpropagation can be seen as resembling a rehabilitation procedure in which the robot is supervised by a therapist in the exploration of the space by means of training examples.

## Experimental results and discussion

In our experiments, we consider a task execution successful when the final layer (*Activator*) activates the output neuronal unit associated with the target area and, consequently, the primitive motor action to remove the target object from the table. A neuronal unit of the Activator layer is considered active if its output value is >0.5. Otherwise, the trial is recorded as an omission. In Tables, we highlight successful attempts in bold values, while omissions are in italicized values.

Each experiment was replicated five times with random weight initialization, and we report the median result in the following tables and text. We considered two test cases for damaging the model: (1) there is no specialization, i.e. LH and RH activate the focus only when the target is in the contralateral area of the attention focus; (2) the RH is specialized and it is able to activate the focus in any area, while the left one can only activate the focus on the right. In both cases, after the initial training phase, the robot learns to execute the task perfectly. Indeed, in a fully “healthy” status, the average likelihood associated with the correct target position is 0.9998 or 0.9999 in both test cases.

### Test case 1: No specialization (control experiment)

In the first case, the plasticity mechanisms are not included in the model: right–left and left–right connections (the dotted lines in Fig. 1) are removed from the neural network; meanwhile, both sides had the same number of neuronal units (eight) and all their connection weights were randomly initialized in the same range [−1,1]. On average, the backpropagation algorithm required 6193 epochs to find the optimal weights during the initial training. After the LH and RH connections are damaged, the results are presented in Tables 1 and 2, respectively. In this test case, we see that the specialization for the spatial attention did not occur as both hemispheres show USN in the contralateral space and results are practically the same. Indeed, we see that the robot with the damaged right hemisphere exhibits USN on the left side, as it is not able to focus all the targets on the left side of attentional space. Even if, in some cases, the likelihood is significantly lower than in the healthy status, no errors were observed. The lower likelihood is common to both LH and RH damages, which achieve very similar results, and it is due to some contribution given by each hemisphere to its own side of the space.

**Table 1:** The LH is damaged: bold values indicate the successful removal of the object in the corresponding area, while italicized values indicate that the area was omitted (i.e. the object was not removed)

Condition A				Condition B			
**1.000**	**0.9957**	*0.0590*	*0.1020*	*0.0590*	*0.2098*	*0.0703*	*0.0577*
**0.9902**	**0.9466**	−*0.0565*	*0.0299*	−*0.0565*	*0.0299*	*0.0004*	*0.1196*

Condition C				Condition D			
**0.6392**	**0.8950**	*0.0747*	*0.1030*	**0.9694**	**0.9122**	**0.6392**	**0.8950**
**0.8778**	**0.9742**	*0.0131*	*0.0944*	**0.9745**	**0.8489**	**0.8778**	**0.9742**

The likelihood of the correct target is also shown

**Table 2:** The RH is damaged: bold values indicate the successful removal of the object in the corresponding area, while italicized values indicate that the area was omitted (i.e. the object was not removed)

Condition A				Condition B			
−*0.1100*	*0.0437*	**0.9752**	**0.9816**	**0.9752**	**0.9816**	**0.9297**	**0.9423**
*0.0954*	−*0.1327*	**0.9513**	**0.9413**	**0.9513**	**0.9413**	**0.9996**	**0.8804**

Condition C				Condition D			
*0.1222*	*0.1050*	**0.9253**	**0.7507**	−*0.1675*	*0.0878*	*0.1222*	*0.1050*
*0.1562*	*0.0258*	**0.8990**	**0.9656**	−*0.0647*	−*0.1278*	*0.1562*	*0.0258*

The likelihood of the correct target is also shown

### Test case 2: Right hemisphere specialization

In this test case, we simulate the right hemisphere specialization for the spatial attention by incorporating the plasticity mechanisms in the network initialization. Indeed, the right hemisphere had a higher number of neuronal units (as reported in Fig. 1) and the connection weights of the left hemisphere were initialized randomly in the range [−0.1, 0.1]. Thanks to this initialization, the model shows some behaviour also described in the real experiment we are replicating. The network was trained by using the same backpropagation algorithm, which required an average of 4740 epochs to learn the optimal connection weights and classify the target positions with an average likelihood of 0.9999. Numerical results are reported in the following Tables 3 and 4.

**Table 3:** Experimental results when the “unspecialized” LH is damaged (control experiment): bold values indicate the successful removal of the object in the corresponding area, while italicized values indicate that the area was omitted (i.e. the object was not removed)

Condition A				Condition B			
**1.000**	**1.000**	**0.5183**	**0.6708**	**0.5183**	**0.6708**	**0.5263**	*0.4730*
**0.9984**	**1.000**	**0.6481**	**0.6655**	**0.6481**	**0.6655**	*0.3323*	*0.4261*

Condition C				Condition D			
**0.8348**	**0.9665**	**0.5182**	*0.4522*	**0.8812**	**0.9831**	**0.8348**	**0.9665**
**0.9557**	**0.7925**	*0.4062*	**0.5116**	**1.0000**	**0.9867**	**0.9557**	**0.7925**

**Table 4:** Experimental results when the “specialized” RH is damaged: bold values indicate the successful removal of the object in the corresponding area, while italicized values indicate that the area was omitted (i.e. the object was not removed)

Condition A				Condition B			
*0.1020*	*0.0187*	**0.6706**	*0.3912*	**0.6706**	*0.3912*	**0.7120**	**0.5777**
*0.0709*	−*0.0051*	**0.5286**	**0.5025**	**0.5286**	**0.5025**	**0.7534**	**0.7057**

Condition C				Condition D			
*0.2539*	−*0.0022*	**0.6639**	**0.6693**	*0.0351*	*0.0351*	*0.2539*	−*0.0022*
*0.1145*	*0.2336*	**0.5569**	**0.5951**	*0.1745*	−*0.0227*	*0.1145*	*0.2336*

From Table 3, we see that only the right side of the spatial attention focus is slightly affected; indeed, problems can be considered minor as only three omissions are registered in condition B, which is the most difficult because all the targets are in the contralateral side of the damage, and two in C. The likelihood is quite high in all cases and, often, it is above 0.4 and near to 0.5 that is the threshold for a successful activation. This confirms that the contribution given by the “unspecialized” LH is weaker than the “specialized” RH.

From Table 4 we see that our experimental results are similar to the findings reported in the work that our experiment is replicating. Indeed, in (Bisiach et al. 1985), authors report more omission (i.e. missed targets) in the contralesional side of the brain lesion, i.e. on the left as the RH is damaged. In particular, we see that the sagittal mid-plane and line of sight contribute significantly to the omissions: when the robot turns its head it is able to remove almost all objects in condition B.

The comparison between results in Tables 3 and 4 clearly suggest that neglect is less severe when LH is damaged, and this is in line with the findings reported in the literature (Mapstone et al. 2003; Monaghan and Shillcock 2004). This difference can be clearly seen both in terms of successful removal of objects, 84.38 and 43.75 %, respectively, when LH and RH are damaged, and of average likelihood, which is 0.737 and 0.345. These numbers confirm that the architecture design led to a specialization of the RH for processing the visual-spatial information from the robot sensors, as its influence to the final result is much stronger than the LH. Indeed, in our experiments, the artificial RH contribution can be estimated as more than 2/3 versus the weaker 1/3 of the artificial LH.

**Fig. 4:**
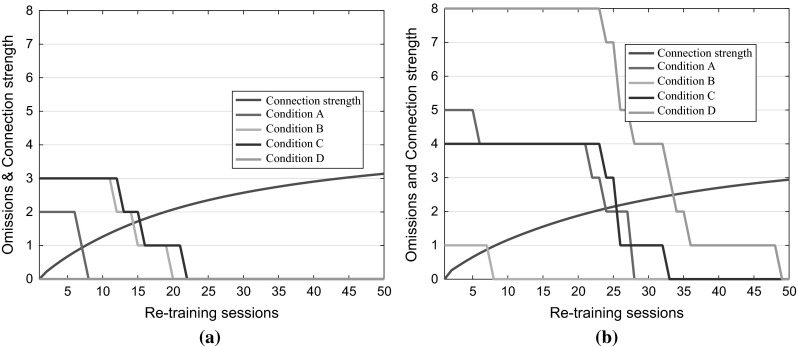
Rehabilitation training results. The figures report omissions and damaged links weights after each session, which comprises 100 epochs of backpropagation. The strength of connection weights is a measure of the recovery speed. **a** The left hemisphere was damaged. **b** The right hemisphere was damaged

Finally, Fig. 4 presents the results of the post-trauma rehabilitation training for both LH (Fig. 4a) and RH (Fig. 4b). Figure 4 reports also the strength of connection weights, which is calculated as the Euclidean distance from the initial condition (i.e. all weights are zero) and measure the speed of the recovery.

By comparing the two plots, we see that the recovery in terms of weights strength is similar between the two hemispheres, and it tends to stabilize around 30 % of the original weights strength. Despite the weaker connections in both cases the robot fully recovers, however faster when the damage is on the LH than RH. In fact, in the case of LH damage, there are no signs of USN after 22 re-training sessions, while in the case of RH damage a full recovery is achieved after 49 sessions. The faster recovery behaviour in case of left damage is frequently reported in the literature (e.g. De Renzi 1982), and it was also observed by Monaghan and Shillcock (2004) who suggest it is evidence of the RH specialization for the elaboration of visual-spatial information.

## Conclusion

This article presented an embodied cognitive robotics approach to the computational modelling of the cognitive dysfunction known as USN. The aim of the study was to introduce and validate a novel model architecture that incorporates the lateral specialization for processing the visual-spatial information. The design of the model hypothesizes plasticity mechanisms that allow the emergence of spatial specialization of the right hemisphere in the experimental task. Finally, we report results of an experimental with the real iCub robot platform that shows behaviours similar to those reported in previous studies with human patients. The present study also highlights some advantages of using an artificial brain embodied in a robotic platform to simulate cognitive dysfunctions.

These results support the use of the cognitive robotics approach to supplement the classical studies to focus on specific parts of the brain and to allow hypothesis and assumptions that are difficult to test in experiments with humans and animals. As an example, we were able to test neglect with LH damage, which is less observed in patients and, moreover, it may imply other problems (e.g. memory, speech, writing, and cognitive processing) that can severely limit patient capabilities to effectively interact (Karnath et al. 2002; Springer and Deutsch 1985), these features make difficult to find subjects with the lesion in the LH available for an experiment. Another advantage is that robots are “tireless” so they can complete the experimental test right after the simulated rehabilitation training, whereas a human patient will be probably tired and this can affect its performance during the test, especially at the beginning of the therapeutic path.

Future work on the model will focus on the relation between USN and body perception, and to further investigate its use in the rehabilitation context.
